# Impact of analysis of risk factors for residual stones after flexible ureteroscopic lithotripsy in patients with upper urinary tract stones

**DOI:** 10.1097/MD.0000000000049518

**Published:** 2026-06-26

**Authors:** Dongdong Fan, Yue Li, Zihui Gao, Yangjun Han

**Affiliations:** a Department of Urology, Peking University First Hospital - Miyun Hospital, Beijing, China; b Department of Urology, The Third Hospital of Hebei Medical University, Shijiazhuang, China.

**Keywords:** DJ stent, preoperative DJ stent placement, stone clearance rate, surgery duration, ureteroscopic lithotripsy

## Abstract

The routine placement of a double-J (DJ) stent before ureteroscopic lithotripsy (URS) remains controversial. This study aims to compare the stone clearance rates between preoperative DJ stent placement and non-placement and to analyze the risk factors associated with residual stones, providing more clinical evidence for preoperative management. A retrospective analysis was conducted on 163 patients who underwent URS for kidney and ureteral stones between April 2023 and October 2024, among which 135 patients met the inclusion criteria. General clinical and perioperative data were collected. The stone clearance rates were compared between the group with preoperative DJ stent placement and the group without DJ stent placement. Univariate and multivariate logistic regression analyses were used to identify risk factors for residual stones after URS. The overall stone clearance rate in patients undergoing URS was 85.19% (115/135). The stone clearance rate in the non-DJ group was 75.51% (37/49), while it was 90.70% (78/86) in the DJ group, showing a statistically significant difference between the 2 groups (*P* = .017). Univariate regression analysis found that the preoperative DJ stent placement (*P* = .021) and surgery duration (*P* = .047) were risk factors for residual stones after URS. Multivariate regression analysis also identified the preoperative DJ stent placement (*P* = .019) and surgery duration (*P* = .039) as independent risk factors for residual stones after URS. Preoperative DJ stent placement resulted in a higher stone clearance rate compared to non-placement during URS. The preoperative DJ stent placement and surgery duration were independent risk factors for residual stones after URS.

## 1. Introduction

The global incidence of urolithiasis ranges from 1 to 20%, making it one of the most common diseases worldwide, with a recurrence rate between 30% and 70%.^[1–4]^ Currently, common treatments for stones include extracorporeal shock wave lithotripsy (ESWL), ureteroscopic lithotripsy (URS), percutaneous nephrolithotomy (PCNL), and the less commonly used open surgery.^[5–7]^ ESWL and URS are more suitable for treating patients with smaller stones, offering less trauma and faster recovery compared to PCNL.^[8,9]^

Although ureteroscopic lithotripsy is minimally invasive, it carries a higher risk of residual stones compared to PCNL. Small residual stones can lead to recurrence and may require repeat surgeries or other adjunct treatments.^[8,10]^ The risk of residual stones is not only influenced by the surgeon’s technique but also by factors such as the patient’s renal anatomy, stone diameter and number, and preoperative comorbidities.^[11–13]^ However, the surgical approach may have a significant impact on stone residuals. Omar et al^[14]^ suggested that preoperative double-J (DJ) stent placement before URS can dilate the ureter, making it easier to insert the ureteral sheath during the second procedure and thus improving stone clearance.

Identifying the risk factors influencing postoperative residual stones is crucial in clinical practice, as it helps to reduce residual stone rates. This study compares the outcomes of URS with and without preoperative DJ stent placement, aiming to provide some references for clinical surgeons when selecting surgical techniques. Additionally, it analyzes the risk factors for residual stones to offer further guidance for clinical management.

## 2. Methods

### 2.1. Study population

**Figure 1.**
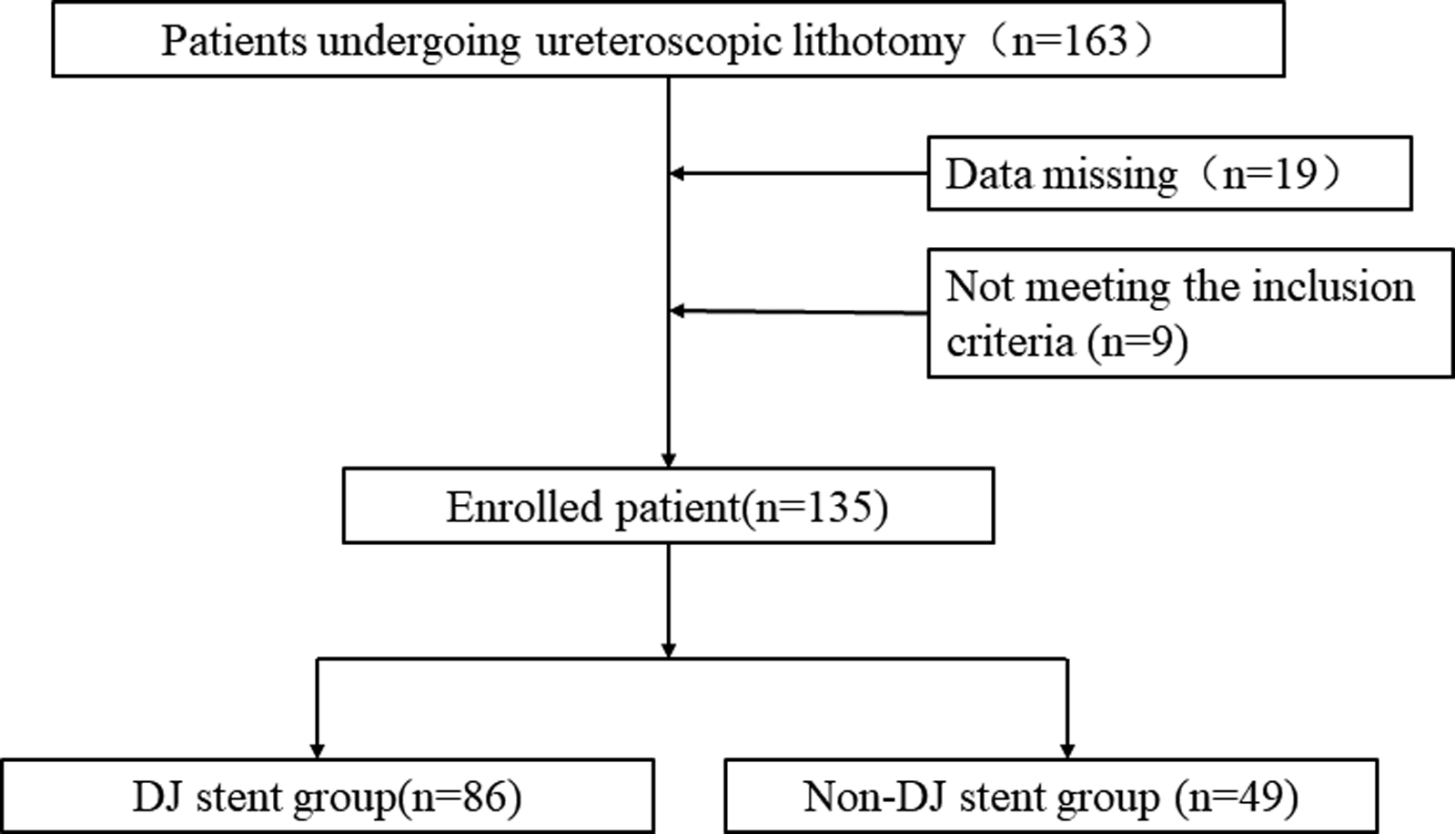
Study flow chart.

A total of 163 patients with kidney stones and upper ureteral stones underwent URS at the Department of Urology, Peking University First Hospital - Miyun Hospital, between April 2023 and October 2024. Among them, 135 patients had complete baseline data and met the inclusion criteria (Fig. 1). Of these, 36.30% (49/135) underwent URS without preoperative DJ stent placement, while 63.70% (86/135) underwent URS with preoperative DJ stent placement. Clinical data, including general clinical characteristics, perioperative data, stone diameter, and postoperative stone clearance rate, were collected.

Stone clearance was defined as no residual stones or the presence of asymptomatic, noninfectious, non-obstructive residual stones ≤ 3 mm, as determined by computed tomography (CT) within one week after surgery. Residual stones were defined as stones that were not cleared.^[15,16]^

The inclusion criteria were listed below, and all of the following conditions needed to be met simultaneously: Kidney or ureteral stones confirmed by preoperative ultrasound, intravenous urography, or urinary system CT; Single stone; Stone diameter ≤ 2 cm; Patients who underwent ureteroscopic lithotripsy; and Complete baseline and follow-up data. Exclusion criteria: Coagulation disorders; and Cardiac dysfunction preventing surgery.

The clinical variables in this study included gender, age, BMI, diabetes, hypertension, coronary heart disease, stone diameter, stone density, stone side, stone location, preoperative DJ stent placement, surgery duration, intraoperative blood loss, blood pressure, and hematological parameters. Residual stones after surgery were used as the outcome to explore the risk factors for residual stones following URS for kidney and ureteral stones. The stone location refers to the anatomical site where the stone forms or lodges in the urinary system, which is categorized into the kidney and the ureter. Its position is determined by CT.

URS without DJ stent placement refers to a single URS procedure completed in one session. URS with preoperative DJ stent placement refers to a two-stage procedure in which a DJ stent is placed during the first surgery, followed by ureteroscopic lithotripsy approximately 2 months later. Surgery duration was defined as the time from the patient entering the operating room until the completion of surgery.

This study was conducted in accordance with the principles of the Declaration of Helsinki (2013 revision) and was approved by the Ethics Committee of Peking University First Hospital - Miyun Hospital. Informed consent was waived for this retrospective analysis.

### 2.2. Surgical technique

After successful anesthesia, the patient was placed in the lithotomy position, and routine disinfection and draping were performed. A size 12F urinary catheter was inserted through the urethra to drain urine, followed by the insertion of the ureteroscope through the urethra into the bladder to inspect the bladder and locate the ureteral orifice. A nickel-titanium alloy guidewire was placed into the ureteral orifice on the affected side, and the ureteroscope was removed. A size 12F access sheath was then left in place, and the ureteroscope was reinserted into the affected ureter under the guidance of the nickel-titanium alloy guidewire. The ureteroscope was advanced to the ureteropelvic junction to inspect the renal pelvis and all renal calyces. Stones were fragmented using a holmium laser, and larger stones were extracted using an N-Gage stone retrieval basket, while smaller stone fragments were left to pass naturally. The renal pelvis and calyces were reexamined to ensure no stones larger than 2mm remained. A hydrophilic guidewire was left in place, and the ureteroscope and sheath were removed. A DJ stent was then placed in the ureter, and the procedure was completed.

### 2.3. Statistical analysis

SPSS version 22.0 was used for statistical analysis. Normally distributed continuous variables were expressed as mean ± standard deviation, while non-normally distributed data were described as median (range). For continuous variables, the *t*-test was used to analyze normally distributed data, and the Mann–Whitney *U* test was used for non-normally distributed data. Fisher exact test was used to analyze categorical variables. Univariate binary logistic regression analysis (*P* < .05) and multivariate logistic regression analysis (*P* < .05) were performed to identify independent risk factors.

## 3. Results

### 3.1. Baseline data of patients

The baseline data of patients undergoing URS are shown in Table 1. A total of 135 patients with kidney and ureteral stones who underwent URS were included in this study, with a mean age of 51.99 ± 12.89 years. The mean stone diameter was 1.15 ± 0.31 cm. Of these patients, 36.30% (49/135) underwent URS without DJ stent placement, while 63.70% (86/135) underwent URS with DJ stent placement. The overall stone clearance rate was 85.19% (115/135).

**Table 1:** Comparison of the efficacy between non-DJ stent group and DJ stent group stone removal using flexible ureteroscopy.

Variable	Total	Non-DJ stent group	DJ stent group	*P* value
Number of patients, n	135	49	86	
Age (yr)	51.99 ± 12.89	48.00 ± 12.61	54.27 ± 12.42	.007
BMI (kg/m^2^)	25.86 ± 3.56	25.51 ± 3.24	26.06 ± 3.69	.397
Gender, n (%)				
Male	87 (64.44)	33 (67.35)	54 (62.79)	.595
Female	48 (35.56)	16 (32.65)	32 (37.21)	
Hypertension, n (%)				
No	88 (65.19)	34 (69.39)	54 (62.79)	.439
Yes	47 (34.81)	15 (30.61)	32 (37.21)	
Diabetes, n (%)				
No	104 (77.04)	38 (77.55)	66 (76.74)	.915
Yes	31 (22.96)	11 (22.45)	20 (23.26)	
CHD, n (%)				
No	125 (92.59)	48 (97.96)	77 (89.53)	.072
Yes	10 (7.41)	1 (2.04)	9 (10.47)	
Side, n (%)				
Left	66 (48.89)	24 (48.98)	42 (48.84)	.884
Right	69 (51.11)	25 (51.02)	44 (51.16)	
Stone location, n (%)				
Kidney	121 (89.63)	42 (85.71)	79 (91.86)	.260
Ureter	14 (10.37)	7 (14.29)	7 (8.14)	
Stone diameter (cm)	1.15 ± 0.31	1.22 ± 0.31	1.12 ± 0.31	.172
Stone density (HU)	864.96 ± 242.42	892.45 ± 327.83	849.30 ± 180.70	.325
Preoperative haemoglobin (g/dL)	138.43 ± 23.54	143.53 ± 20.61	135.52 ± 24.48	.059
Preoperative white blood cells (×10^9^/L)	7.14 ± 6.96	6.52 ± 1.57	7.49 ± 8.62	.446
Preoperative creatinine (mg/dL)	78.64 ± 44.21	82.15 ± 58.66	76.71 ± 33.03	.504
Preoperative blood sodium (mmol/L)	140.24 ± 1.72	140.21 ± 1.57	140.26 ± 1.79	.869
Preoperative blood potassium (mmol/L)	7.34 ± 3.18	4.76 ± 5.19	8.78 ± 0.40	.827
Preoperative systolic pressure (mm Hg)	127.41 ± 8.14	126.69 ± 7.11	127.18 ± 8.61	.447
Preoperative diastolic pressure (mm Hg)	77.74 ± 6.03	78.22 ± 6.26	77.76 ± 5.84	.487
Preoperative blood calcium (mmol/L)	2.37 ± 6.03	2.35 ± 0.15	2.28 ± 0.36	.592
Postoperative haemoglobin (g/dL)	131.70 ± 19.37	133.86 ± 19.21	130.48 ± 19.25	.335
Postoperative white blood cells (×10^9^/L)	9.51 ± 7.92	8.97 ± 3.77	9.82 ± 9.47	.552
Postoperative creatinine (mg/dL)	81.77 ± 44.08	85.82 ± 58.64	79.40 ± 32.02	.424
Postoperative blood sodium (mmol/L)	141.48 ± 2.53	141.56 ± 2.33	141.44 ± 2.63	.800
Postoperative blood potassium (mmol/L)	3.97 ± 0.39	3.98 ± 0.35	3.97 ± 0.41	.827
Postoperative blood calcium (mmol/L)	2.23 ± 0.17	2.23 ± 0.13	2.22 ± 0.19	.903
Surgery duration (min)	89.87 ± 43.38	89.53 ± 41.18	90.06 ± 44.36	.947
Intraoperative blood loss (mL)	5.18 ± 8.64	5.37 ± 7.93	5.07 ± 8.98	.849
Hospitalization times (d)	6.70 ± 2.05	6.82 ± 1.69	6.64 ± 2.22	.826
Stone clearance, n (%)				
No	20 (14.81)	12 (24.49)	8 (9.30)	.017
Yes	115 (85.19)	37 (75.51)	78 (90.70)	

BMI = body mass index, CHD = coronary artery heart disease, DJ = double-J.

### 3.2. Impact of preoperative DJ stent placement versus non-placement on stone clearance rates

The clinical data of patients in the DJ stent group and the non-DJ stent group are shown in Table 1. The stone clearance rate in the non-DJ stent group was 75.51% (37/49), while it was 90.70% (78/86) in the DJ stent group, with a statistically significant difference between the 2 groups (*P* = .017). The mean age of the non-DJ stent group was 48.00 ± 12.61 years, while the DJ stent group had a mean age of 54.27 ± 12.42 years, with a statistically significant difference between the 2 groups (*P* = .007). The mean stone diameter in the non-DJ stent group was 1.22 ± 0.31 cm, and in the DJ stent group, it was 1.12 ± 0.31 cm, with no statistically significant difference between the 2 groups (*P* = .172).

### 3.3. Analysis of risk factors for residual stones after ureteroscopic lithotripsy

The clinical data of patients in the residual stone group and the stone clearance group are shown in Table 2. The proportion of patients without preoperative DJ stent placement in the residual stone group was 60.00% (12/20), compared to 32.17% (37/115) in the stone clearance group, with a statistically significant difference between the 2 groups (*P* = .017). The mean preoperative white blood cell count in the residual stone group was 10.04 ± 16.85 × 10^9^/L, while it was 6.63 ± 1.82 × 10^9^/L in the stone clearance group, with a statistically significant difference (*P* = .043). The mean surgery duration in the residual stone group was 108.15 ± 36.46 minutes, while it was 86.66 ± 43.57 minutes in the stone clearance group, with a statistically significant difference (*P* = .042).

**Table 2:** Comparison between the stone remnant group and the stone clearance group after flexible ureteroscopy.

Variable	Stone remnant group	Stone clearance group	*P* value
Number of patients, n	20	115	
Age (yr)	50.95 ± 8.68	52.17 ± 13.45	.699
BMI (kg/m^2^)	25.43 ± 2.38	25.94 ± 3.72	.560
Gender, n (%)			
Male	14 (70.00)	73 (63.48)	.574
Female	6 (30.00)	42 (36.52)	
Hypertension, n (%)			
No	14 (70.00)	74 (64.35)	.062
Yes	6 (30.00)	41 (35.65)	
Diabetes, n (%)			
No	17 (85.00)	87 (75.65)	.359
Yes	3 (15.00)	28 (24.35)	
CHD, n (%)			
No	19 (95.00)	106 (92.17)	.656
Yes	1 (5.00)	9 (7.83)	
Side, n (%)			
Left	14 (70.00)	52 (45.22)	.102
Right	6 (30.00)	63 (54.78)	
Stone location, n (%)			
Kidney	16 (80.00)	105 (91.30)	.126
Ureter	4 (20.00)	10 (8.70)	
Preoperative DJ stent placement, n (%)			
No	12 (60.00)	37 (32.17)	.017
Yes	8 (40.00)	78 (67.83)	
Stone diameter (cm)	1.20 ± 0.37	1.14 ± 0.29	.516
Stone density (HU)	907.50 ± 168.02	857.57 ± 255.01	.401
Preoperative haemoglobin (g/dL)	141.45 ± 12.65	137.90 ± 24.88	.539
Preoperative white blood cells (×10^9^/L)	10.04 ± 16.85	6.63 ± 1.82	.043
Preoperative creatinine (mg/dL)	75.10 ± 20.07	79.28 ± 47.15	.701
Preoperative blood sodium (mmol/L)	140.54 ± 1.93	140.19 ± 1.66	.418
Preoperative blood potassium (mmol/L)	4.06 ± 0.22	4.34 ± 3.43	.718
Preoperative systolic pressure (mm Hg)	126.50 ± 5.36	127.57 ± 8.50	.594
Preoperative diastolic pressure (mm Hg)	79.35 ± 4.35	77.46 ± 6.21	.200
Preoperative blood calcium (mmol/L)	2.34 ± 0.10	2.38 ± 0.32	.619
Postoperative haemoglobin (g/dL)	132.25 ± 16.03	131.61 ± 19.84	.893
Postoperative white blood cells (×10^9^/L)	11.88 ± 17.24	9.10 ± 4.31	.152
Postoperative creatinine (mg/dL)	82.05 ± 23.21	81.72 ± 46.74	.975
Postoperative blood sodium (mmol/L)	141.74 ± 3.05	141.44 ± 2.41	.627
Postoperative blood potassium (mmol/L)	3.96 ± 0.21	3.97 ± 0.41	.928
Postoperative blood calcium (mmol/L)	2.23 ± 0.09	2.38 ± 0.32	.940
Surgery duration (min)	108.15 ± 36.46	86.66 ± 43.57	.042
Intraoperative blood loss (mL)	4.90 ± 6.13	5.23 ± 8.99	.878
Hospitalization times (d)	7.00 ± 1.80	6.65 ± 2.08	.489

BMI = body mass index, CHD = coronary artery heart disease, DJ = double-J.

Univariate regression analysis identified preoperative DJ stent placement (*P* = .021) and surgery duration (*P* = .047) as risk factors for residual stones after URS. Multivariate regression analysis also confirmed that preoperative DJ stent placement (*P* = .019) and surgery duration (*P* = .039) were independent risk factors for residual stones after URS (Table 3).

**Table 3:** The univariate and multivariate regression analysis of stone remnant of kidney and ureteral stones treated with flexible ureteroscopy.

	Univariate analysis		Multivariate analysis	
Characteristic	HR (95% CI)	*P* value	HR (95% CI)	*P* value
Age	0.993 (0.957-1.030)	.696		
BMI	0.959 (0.832-1.104)	.556		
Gender	1.342 (0.480-3.756)	.575		
Hypertension	1.293 (0.462-3.620)	.625		
Diabetes	1.824 (0.497–6.686)	.365		
CHD	1.613 (0.193–13.479)	.659		
Side	2.250 (0.836–6.052)	.108		
Stone location	0.381 (0.107–1.361)	.137		
Preoperative DJ stent placement	3.162 (1.191-8.395)	.021	3.317 (1.218–9.033)	.019
Stone diameter	1.741 (0.331–9.154)	.512		
Stone density	1.001 (0.999-1.003)	.400		
Preoperative hemoglobin	1.007 (0.985–1.030)	.535		
Preoperative white blood cells	1.047 (0.977–1.122)	.195		
Preoperative creatinine	0.997 (0.984–1.011)	.701		
Preoperative blood sodium	1.123 (0.849–1.486)	.415		
Preoperative blood potassium	0.946 (0.671–1.334)	.753		
Preoperative systolic pressure	0.984 (0.926–1.045)	.591		
Preoperative diastolic pressure	1.049 (0.975–1.128)	.202		
Preoperative blood calcium	0.547 (0.050–5.960)	.62		
Surgery duration	1.010 (1.000–1.020)	.047	1.011 (1.001–1.022)	.039

BMI = body mass index, CHD = coronary artery heart disease, CI = confidence interval, DJ = double-J, HR = hazard ratio.

## 4. Discussion

RIRS, ESWL, and PCNL are the main treatment options for kidney stones.^[17]^ With advancements in technology, significant improvements have been made in optics, mechanics, and laser technology, leading to the widespread application of ureteroscopic lithotripsy. This study explores whether preoperative placement of a DJ tube is more beneficial for stone clearance in flexible ureteroscopy and simultaneously analyzes the risk factors for postoperative residual stones.

The debate over whether preoperative stent placement is necessary before ureteroscopic lithotripsy has persisted for many years.^[18]^ Some clinicians believe that preoperative stent placement increases the success rate and stone clearance rate of the surgery,^[19]^ while others argue that stent placement is not necessary for kidney stones.^[20]^ In this study, the overall stone clearance rate after ureteroscopic lithotripsy was 85.19% (115/135), with a clearance rate of 75.51% (37/49) in the non-DJ group and 90.70% (78/86) in the DJ group, showing a statistically significant difference between the 2 groups (*P* = .017). Dessyn et al^[21]^ also found that the stone clearance rate was higher when a DJ stent was placed before ureteroscopic lithotripsy, consistent with our findings. However, Pan et al^[18]^ studied single-kidney patients and found that preoperative stent placement did not significantly improve the stone clearance rate, though it may help protect renal function postoperatively. Different centers have different findings regarding postoperative stone clearance rates, and our study suggests that preoperative DJ stent placement can improve stone clearance.

Although there is still controversy over whether preoperative DJ stent placement improves stone clearance, there is a consensus that stent placement should be performed in patients with renal colic or sepsis before subsequent ureteroscopic lithotripsy.^[22,23]^ DJ stent placement can relieve obstruction, effectively alleviate pain caused by renal colic, and control sepsis.^[24,25]^ Although preoperative DJ stent placement has several advantages, it may also lead to side effects such as lower back pain, urinary difficulty, frequent urination, or hematuria.^[25]^

This study also analyzed the risk factors for residual stones and found that preoperative DJ stent placement (*P* = .019) and surgery duration (*P* = .039) were independent risk factors for residual stones after ureteroscopic lithotripsy. Netsch et al^[26]^ compared patients with and without preoperative ureteral stent placement and found a significantly higher stone-free rate in patients with stent placement (95.1% vs 86.7%, *P* = .013). Pan et al^[18]^ also found that the incidence of residual stones was higher in patients without preoperative DJ stent placement, particularly in lower pole stones. Several factors may contribute to the higher stone clearance rate with preoperative DJ stent placement. The stent can continuously dilate the ureter, reduce intraoperative ureteral narrowing or spasms, enhance the flexibility of the ureteroscope, and improve the visual field, thereby facilitating complete stone clearance. Ureteral edema caused by stone impaction or intraoperative trauma may impair stone visibility and surgical manipulation, while preoperative DJ stent placement can alleviate edema and improve intraoperative conditions. Therefore, our study suggests that preoperative DJ stent placement is a beneficial clinical decision.

Previous studies have found that longer surgery duration is associated with postoperative infections and other complications, but our study revealed that longer surgery duration may also increase the risk of residual stones.^[27–29]^ Komeya et al^[30]^ found that prolonged surgery duration in ureteroscopic lithotripsy reduced stone fragmentation efficiency, while patients with shorter surgery times had higher stone-free rates. This finding aligns with the conclusions of our study. Several factors may explain the increased residual stone rate with longer surgery duration: Prolonged surgery may lead to tissue edema, resulting in poor visibility and difficulty in locating and removing stones; Longer surgery times may increase surgeon fatigue, reducing precision and affecting complete stone removal; and Prolonged procedures may cause increased intrarenal pressure, especially during continuous irrigation, which can interfere with stone visibility and extraction. Therefore, optimizing the surgical process and minimizing surgery duration, while ensuring patient safety, are important measures to improve stone clearance rates.

Our study has several limitations. This study is a retrospective study, and some data on influencing factors related to postoperative residual stones are missing. The missing data may include some detailed clinical information, such as records of the patient’s dietary structure within a specific period before surgery (e.g., daily calcium and oxalate intake), monitoring data of activity levels in the short term after surgery, and some special laboratory test results (e.g., the precise concentration of certain specific ions in urine). In addition, some potential influencing factors, such as the patient’s genetic factors (specific gene polymorphisms related to stone formation) and long-term medication history (certain drugs that may affect urine composition), may also be missing. Additionally, this was a single-center study, which may introduce bias. We aim to establish a multi-center prospective cohort study to increase the sample size and more accurately analyze the risk factors for residual stones after ureteroscopic lithotripsy.

## 5. Conclusion

Our study found that preoperative DJ stent placement resulted in a higher stone clearance rate after ureteroscopic lithotripsy. Additionally, multivariate analysis revealed that preoperative DJ stent placement and surgery duration were independent risk factors for residual stones after ureteroscopic lithotripsy. Specifically, selecting appropriate stone removal techniques and controlling surgery duration can significantly reduce the incidence of postoperative residual stones. In clinical practice, optimizing preoperative and intraoperative strategies based on individual patient conditions will help improve surgical success rates and reduce the risk of residual stones.

## Author contributions

**Conceptualization:** Dongdong Fan, Yue Li, Yangjun Han.

**Data curation:** Dongdong Fan, Yangjun Han.

**Formal analysis:** Yangjun Han.

**Investigation:** Dongdong Fan.

**Methodology:** Zihui Gao, Yangjun Han.

**Software:** Yue Li.

**Writing – original draft:** Dongdong Fan, Yue Li, Zihui Gao, Yangjun Han.

**Writing – review & editing:** Dongdong Fan, Yue Li, Zihui Gao, Yangjun Han.
